# *Astragalus membranaceus* and *Cinnamomum cassia* Stimulate the Hair Follicle Differentiation-Related Growth Factor by the Wnt/β-Catenin Signaling Pathway

**DOI:** 10.3390/cimb45110541

**Published:** 2023-10-26

**Authors:** Mi Hye Kim, Seong Chul Jin, Hee Kyung Baek, Woong Mo Yang

**Affiliations:** 1 College of Korean Medicine, Woosuk University, Wanju 55338, Republic of Korea; kimmh526@woosuk.ac.kr; 2 Department of Convergence Korean Medical Science, College of Korean Medicine, Kyung Hee University, Seoul 02447, Republic of Korea; misterjin23@khu.ac.kr (S.C.J.); bhkbjk@khu.ac.kr (H.K.B.)

**Keywords:** YH complex, *Astragalus membranaceus*, *Cinnamomum cassia*, hair loss, Wnt/β-catenin signaling pathway

## Abstract

*Astragalus membranaceus* and *Cinnamomum cassia* are used as spices and flavorful ingredients, or medicinal herbs with pharmacological effects. In this study, the hair-growth-promoting effects of the YH complex, a newly developed formula consisting of *membranaceus* and *C*. *cassia*, are investigated with the prediction of its molecular mechanism. The target gene of the YH complex was about 74.8% overlapped with the gene set of ‘Hair growth’ on the GO Biological Process database. The oral administration of the YH complex promoted hair regrowth and increased hair-shaft thickness in depilated hair loss mice. In addition, the anagen/telogen hair follicle ratio was significantly increased by the YH complex. The growth factors affecting the growth of hair follicles were dose-dependently increased by treatment with the YH complex. The Wnt/β-catenin signaling pathway expressions in skin tissues were apparently increased by the administration of the YH complex. In conclusion, the YH complex consisting of *A*. *membranaceus* and *C*. *cassia* induced hair follicle differentiation and preserved the growing-anagen phase by increasing growth factors and the Wnt/β-catenin signaling pathway, leading to the restoration of hair loss. The YH complex can be a remedy for hair loss diseases, such as alopecia areata, androgenetic alopecia, telogen effluvium, and chemotherapy-induced alopecia.

## 1. Introduction

The scalp of human beings is covered with about 100,000 hairs, protecting the scalp and brain from external stimuli, such as physical collision, changes in temperature and humidity, or ultraviolet light from the sun [1]. The structure of hair consists of two parts: the hair shaft and hair follicle [2]. The upper part of the hair, which can be observed above the skin, is the hair shaft. The hair follicle, the lower part of the hair, regulates hair growth from a complex interaction between hormones, neuropeptides, and immune cells [3]. Per day, hair grows at a rate of 0.37 mm, and fewer than 75 hairs are lost. Hair loss is determined when the average loss is more than 100 hairs per day [4]. Though hair loss is a common symptom, there are some factors that contribute to it, such as hereditary or hormonal (intrinsic factors), as well as the use of medications, hair products, or pulled hair styles (extrinsic factors) [5].

Hair loss, except for rare congenital hair defects and scarring alopecia, reflects aberrations of hair follicle cycling [6]. The hair follicle cycle consists of three phases: anagen, catagen, and telogen [7]. Anagen is the growth phase, lasting 2 to 6 years on the human scalp. After 2 to 3 weeks of catagen, the hair growth state enters a rest period, which is called telogen [8]. As the hair length is determined by the duration of the anagen phase, the hair follicle cycle plays an important role in hair growth [9]. In addition, hair can indicate a person’s age, sexuality, religion, or job, and can identify personality traits by the way it is cared for and groomed [10]. As hair loss progresses, people can feel like they look old or unattractive and fear social exclusion [11]. Patients suffering from alopecia tend to have lower self-confidence or self-esteem, which leads to heightened self-consciousness [12]. In addition, most alopecia patients have depressive symptoms, implying that hair has symbolic meaning in society [13].

Finasteride and minoxidil are two of the most commonly used treatments for hair loss, which have been proven to be effective in promoting hair growth [14]. Finasteride, a type-II 5α-reductase inhibitor, has been approved by the US Food and Drug Administration (FDA) for the treatment of male pattern baldness. It works by blocking the conversion of testosterone to dihydrotestosterone (DHT), which is responsible for shrinking hair follicles and leading to hair loss [15]. Similarly, minoxidil, a potassium channel opener, was initially developed as a vasodilator for the treatment of high blood pressure [16]. However, this treatment also has a number of side effects, including decreased libido, impotence, and itching. Daily oral finasteride treatment in androgenetic alopecia patients exhibited sexual dysfunction symptoms, including erectile dysfunction and a low libido, compared to the placebo group [17]. Additionally, the topical application of minoxidil has been reported to induce irritation and allergic contact dermatitis, pruritus, scalp irritation, and facial hypertrichosis [18], whereas the systemic side effects of taking the oral form of minoxidil were noted to increase heart rate, weight gain, hirsutism, hypertrichosis, and lower-extremity edema [19]. Furthermore, in rare cases, they may cause an allergic reaction, leading to severe skin irritation, redness, and swelling. So, it is important to make efforts to reduce the side effects [20].

The use of natural products in medicine has been an area of interest for researchers and healthcare professionals alike. In recent years, there has been a growing recognition of the potential advantages of natural products as adjuvants to the existing treatments [21]. The use of natural products as adjuvants has several benefits, including improved patient outcomes, reduced side effects, and increased patient satisfaction. The use of natural products as an alternative to finasteride and minoxidil is an area of growing interest. Many natural ingredients, such as saw palmetto, biotin, and niacin, have been shown to have potential for promoting hair growth and preventing hair loss.

The YH complex is a herbal medicine developed for alleviating hair loss and promoting hair regrowth. It consists of the radix of *Astragalus membranaceus* and cortex of *Cinnamomum cassia*. *A*. *membranaceus*, also known as Huang Qi, is a species of legume that is commonly used in traditional medicine, as well as being a food ingredient [22]. It has been used to treat various health conditions, including fatigue, anemia, and heart disease [23]. Studies have shown that *A*. *membranaceus* has multiple pharmacological effects, including immunomodulatory, anti-inflammatory, antioxidant, and antitumor activities [24]. *C*. *cassia*, commonly known as true cinnamon, is widely used as a spice and flavor ingredient in food and beverages [25]. It has been used in traditional medicine for the treatment of various health conditions, including digestive problems, colds, and menstrual discomfort [26]. Studies have shown that *C*. *cassia* has multiple pharmacological effects, including antioxidant, anti-inflammatory, and antidiabetic activities [27]. Despite the reported efficacy of these herbs, there is limited scientific evidence to support their effectiveness in promoting hair growth. The exact mechanisms by which they exert their effects remain unknown. It is imperative to conduct rigorous scientific studies to establish the efficacy and safety of these herbs for the treatment of hair growth.

In this study, the efficacy of the YH complex, a combination of *A*. *membranaceus* and *C*. *cassia*, is investigated in regard to hair growth and the related pathways. The results of this study contribute to the body of knowledge on the use of herbal remedies to supplement hair growth and help to inform the future research in this field.

## 2. Materials and Methods

### 2.1. Network Analysis

The YH complex network was constructed with *A*. *membranaceus* radix and *C*. *cassia* cortex compounds based on the previous method [28]. Compounds of the YH complex were collected based on the TM-MC database (https://informatics.kiom.re.kr/compound/search.do (accessed on 17 January 2023)) (Tables S1 and S2). The total compounds, each gene with co-occurrence for each compound, were collected through PubChem (https://pubchem.ncbi.nlm.nih.gov/) (Tables S3 and S4). Among the genes related to the compound (Tables S5–S7), redundancies were removed, and the network components were selected based on the genes with high reliability (score 0.7) through the STRING database (http://www.string-db.org/). Additionally, we selected genes expressed in hair follicles through the HUMANBASE database (https://hb.flatironinstitute.org/) (Table S8). Finally, we created a network of YH complex via Cytoscape (3.9.1) using selected genes.

### 2.2. Functional Enrichment Analysis on the GO Biological Process Database

Biological pathways associated with the network of the YH complex were investigated by Cytoscape functional enhancement assay. The pathways and targets of the network were analyzed and classified by the gene ontology (GO) process and Kyoto Encyclopedia of Genes and Genomes (KEGG) pathway databases. Functions of the YH complex were expressed and configured based on FDR value.

### 2.3. Sample Preparation

The YH complex was obtained from COSMAX NS, INC. (Seongnam, Republic of Korea). Briefly, dried *C*. *cassia* cortex and *A*. *membranaceus* radix were extracted in water. After filtering, the remaining extract was concentrated and powdered with maltodextrin by spray-drying method. The identification of YH complex was conducted with the standards of cinnamic acid and calycosin-7-O-glucoside (Figure S1). All voucher specimens were stored at COSMAX NS, INC.

### 2.4. Animal Experiments

The experimental procedures were approved by the Committee on Care and Use of Laboratory Animals of the Kyung Hee University (KHSASP-22-478). Six-week-old male C57BL/6 mouse were obtained from DBL Co. (Eumseong, Republic of Korea). All mice were fed water and mouse chow ad libitum, with 12 h light and 12 h dark cycle at a temperature of 22 ± 2 °C and 55 ± 10% humidity. The mice were randomly divided into five groups (eight mice per group): NOR (normal mice without hair removal) and HR (hair removal mice), Fina (hair removal mice with finasteride 1 mg/kg oral administration), YH50 (hair removal mice with YH complex 50 mg/kg oral administration), and YH150 (hair removal mice with YH complex 150 mg/kg oral administration). The back skins of mice were shaved using an electric clipper and Niclean shaving cream (Ildong Pharmaceuticals, Seoul, Republic of Korea). After 1 week of accumulation, 100 μL of vehicle (distilled water) was administered to NOR and HR groups, 100 μL of finasteride to the Fina group, and the YH complex to YH50 and YH150 groups. The treatment was performed five days a week for 3 weeks. All procedures were conducted in accordance with the regulations for the Care and Use of Laboratory Animals of the Kyung Hee University.

### 2.5. Morphological and Dermatological Analysis

At 21 days after oral administration, all animals were anesthetized by intraperitoneal injection with Zoletil:Rompun 1:1 mixture and photographs of each group were taken. The assessment of hair growth and density per unit area were measured by dermoscopy with ×10 magnification. The dermoscopic images from dermal skin were obtained from a Smartscope (#KJMSF-02, ver. 1.53K, Kangjin Technology, Ltd., Seoul, Republic of Korea). Then, the relative hair densities in dermoscopic images were analyzed by ImageJ software (ver. 1.4.3.x., U. S. National Institutes of Health, Bethesda, MD, USA). To quantify the hair area, all images were converted to a grayscale format, then adjusted using the threshold function for contrasting between the hair and the surrounding area. Subsequently, the hair area and density were determined.

### 2.6. Scanning Electron Microscopy (SEM)

Hair samples were collected from skin tissues after sacrifice. Then, all the hair samples were washed separately using phosphate-buffered solution (PH 7.4) and vortexed for about three to five minutes. After the washing process, the hair samples were dried in an oven at a temperature of 40–50 °C for about 30 min. Hair samples were attached to the carbon tape in a high vacuum coated with platinum. The morphologies of the coated samples were examined using field emission gun scattering electron microscopy (10 kv, Inspect F50, FEI).

### 2.7. Histology

The dorsal skin tissues were collected from each mouse after sacrifice and immediately fixed in 4% formaldehyde for 24 h. After dehydration, the skin tissues were embedded in paraffin. The paraffin-embedded tissues were cut into 5 μm thickness and placed onto slide glasses. The sections for histological examination were stained with hematoxylin and eosin solution. Leica Application Suite Microscope Software version 4.12.0.86 (Leica Microsystems Inc., Deerfield, IL, USA) was used to obtain digital images at a magnification of ×200. Following image acquisition, we counted the number of anagen- and telogen-like hair follicles. The relative proportion and ratio of Anagen-like hair follicles/telogen-like hair follicles were quantified.

### 2.8. Immunohistochemistry

The 5 μm sectioned skin tissues were treated with 3% H_2_O_2_ for 30 min to block endogenous peroxidase after deparaffinization. After blocking, sections were incubated with primary antibody keratin (Ks)6 (1:500, Santa Cruz Biotechnology, Santa Cruz, CA, USA) and Ks10(1:500, Biolegend, San Diego, CA, USA) overnight. Biotinylated horse anti-rabbit IgG (1:200, Vector laboratories, Newark, CA, USA) and biotinylated horse anti-rabbit IgG (1:200, Vector laboratories) were treated for 1 h at room temperature. After washing using PBS, sections were incubated with an Elite avidin/biotinylated enzyme complex kit (Vector laboratories). After an hour, peroxidase conjugates were subsequently visualized with diaminobenzidine (DAB; Sigma-Aldrich; Merck Millipore, Darmstadt, Germany) solution and hematoxylin. A Leica Application Suite Microscope (Leica Microsystems Inc.) was used to obtain digital images. The magnification was ×200.

### 2.9. Reverse Transcription Polymerase Chain Reaction

The total RNA sample from the skin tissues was obtained by extraction with the TRIzol method. According to the manufacturer’s instruction, complementary DNA (cDNA) was synthesized at 45 °C for 60 mins and then at 95 °C for 5 mins. After that, a reverse transcription polymerase chain reaction (RT-PCR) was carried out with the Taq polymerase and specific mRNA primers. Using a 1% agarose gel, the PCR products were separated. The resulting bands were visualized with a computerized densitometry system (ImageJ).

### 2.10. Western Blotting Analysis

Proteins from samples were extracted from C57BL/6 mice. For use in Western blotting, the skin tissues were homogenized in tissue protein extraction buffer (Thermo Scientific, Rockford, IL, USA) with protease inhibitor cocktail tablets (Roche, Mannheim, Germany). After vortexing, the lysates were pelleted by centrifuge at 17,000× *g* for 10 min and 4 °C. Equal amount of protein (10 μg) was subjected to 12.5% polyacrylamide gel electrophoresis and then transferred to polyvinylidene fluoride membranes. PVDF membranes were treated with primary antibodies at 4 °C overnight (β-actin, bone morphogenetic protein (BMP)-2, transforming growth factor (TGF)-β1, Dickkopf (DKK)1, Wingless-related integration site (Wnt)3a and β-catenin; Cellsignal, Danvers, CA, USA, 1:1000 dilutions in TBS-T). After three washes, the membranes were incubated with anti-mouse and anti-rabbit horseradish peroxidase-coupled secondary antibodies (Cellsignal, 1:3000 dilution in TBS-T) for 1 h at room temperature. Proteins were detected by an enhanced chemiluminescence detection reagent (AbClon, Seoul, Republic of Korea) by a chemiluminescence imaging system (Amersham, Uppsala, Sweden). The relative density of each expression was normalized to β-actin.

### 2.11. Statistical Analysis

All results were indicated as the means ± standard error of the mean (S.E.M.). For comparison with data, one-way ANOVA followed by Tukey’s multiple tests was used in these groups. The *p* values were given as follows: * *p* < 0.05, ** *p* < 0.01, and *** *p* < 0.001 were considered statistically significant.

## 3. Results

### 3.1. Investigation of the Association of YH Complex and Hair Growth through Network Construction

**Figure 1:**
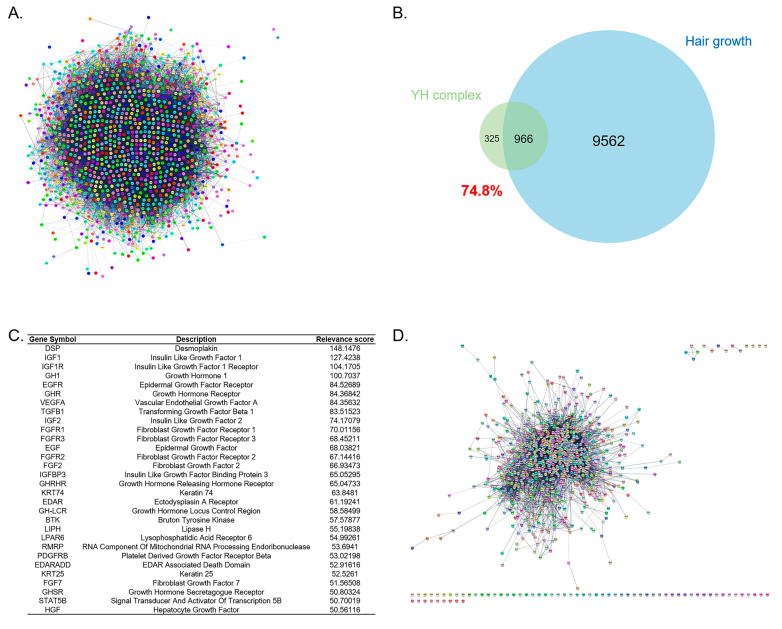
Network analysis of the combination of *Astragalus membranaceus* and *Cinnamomum cassia*, consisting of YH complex. (**A**) Whole network of the YH complex-targeted 1291 genes containing 1271 nodes and 30,230 edges. Blue, cooccurrence; light green, textmining; sky blue, databases; purple, experiments; black, coexpression; green, neighborhood; red, fusion. (**B**) Venn diagram of the YH complex and hair growth gene set. (**C**) Top 30 target genes of ‘Hair growth’ gene set. (**D**) Network of 966 common genes of the YH complex and the hair growth gene set.

The whole network of the YH complex was obtained from 1971 target genes and 1049 target genes of *C*. *cassia* and *A*. *membranaceus*, respectively. After removing duplicates and conversing into protein query name, a total of 1291 target genes were collected for the YH complex network, containing 1271 nodes and 30,230 edges (Figure 1A). To investigate the association of the YH complex and hair growth, the common genes of 1291 targeted genes of YH complex and 10,528 targeted genes of ‘Hair growth’ were counted. Eventually, 966 intersection genes were identified; thus, the YH complex had 74.8% overlapping correlation (Figure 1B). *DSP*, *IGF1*, *IGF1R*, *GH1*, *EGFR*, *GHR*, *VEGFA*, *TGFB1*, *IGF2*, *FGFR1*, *FGFR3*, *EGF*, *FGFR2*, *FGF2*, *IGFBP3*, *GHRHR*, *KRT74*, *EDAR*, *GH-LCR*, *BTK*, *LIPH*, *LPAR6*, *RMRP*, *PDGFRB*, *EDARADD*, *KRT25*, *FGF7*, *GHSR*, *STAT5B*, and *HGF* were found to be the top 30 target genes with high relevance scores (Figure 1C). Among those, a network including 986 nodes and 10,490 edges of common genes on the YH complex and the ‘Hair growth’ network was constructed (Figure 1D).

### 3.2. Prediction of Significant Pathway by Functional Enrichment Analysis of the Target Genes of YH Complex and Hair Growth

**Figure 2:**
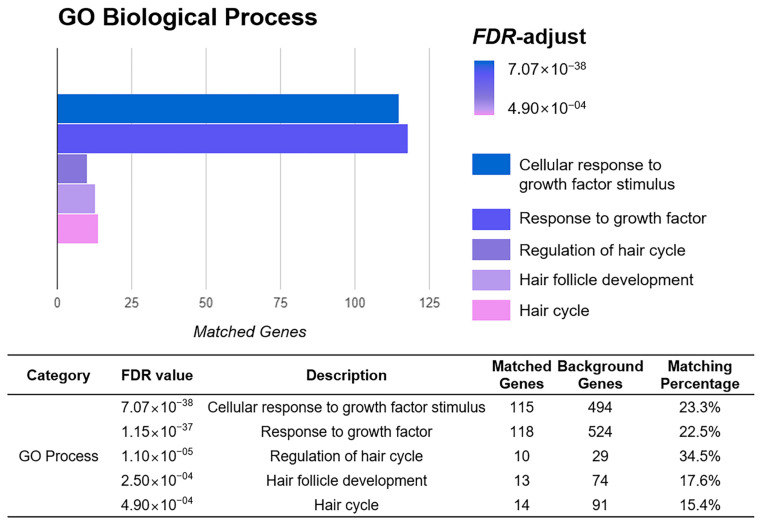
Predicted functional biological terms of common genes of YH complex and hair growth gene set. The target pathway was listed from the GO Biological Process database. According to the score of FDR value, the description was listed and matching percentage was calculated.

Functional enrichment analysis was carried out using 966 intersecting genes between the YH complex and ‘Hair growth’ network. Based on the highest FDR-value, ‘Cellular response to growth factor stimulus’, ‘Response to growth factor’, ‘Regulation of developmental growth’, ‘Regulation of hair cycle’, ‘Hair follicle development’, ‘Hair cycle’, and ‘Hair follicle morphogenesis’ were listed on the GO Biological Process database. According to the UniProt Keywords, ‘Growth factor’ was founded. In addition, KEGG Pathways database showed that ‘PI3K-Akt signaling pathway’, ‘MAPK signaling pathway’, and ‘Growth hormone synthesis, secretion and action’ were related to the functional potential pathway of the YH complex on hair growth (Figure 2).

### 3.3. Effects of YH Complex on Morphological Changes and Hair Density in Hair Removal Model

**Figure 3:**
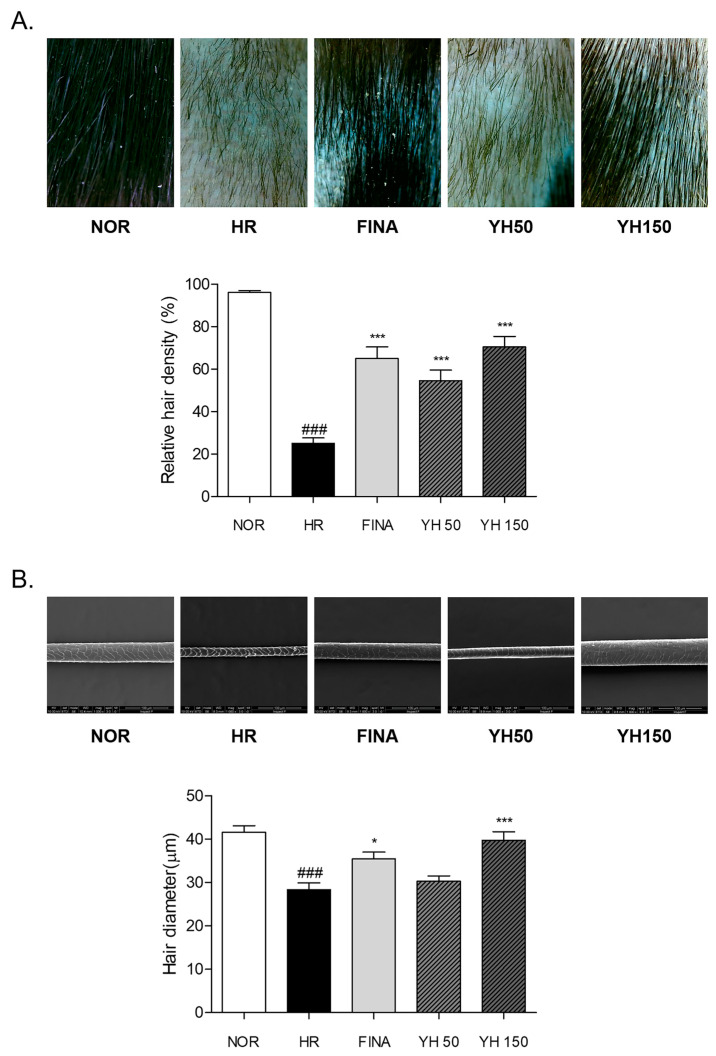
Hair regrowth rate of dorsal skin tissues. (**A**) Skin color and hair density monitored by dermoscope. The relative hair densities per unit area were measured. (**B**) Scanning electron microscopy (SEM) analysis of hair shaft. The hair diameter of hair shaft was calculated. Results are presented as mean ± standard error of the mean. ^###^ *p* < 0.001 vs. NOR group; * *p* < 0.05 and *** *p* < 0.001 vs. HR group. HR, hair removal; FINA, finasteride; YH, YH complex.

We tested the hair-growth-promoting activity of the YH complex. In morphological observation, the following results were obtained from visually observing the mouse’s hair growth state after hair removal. In the YH 150 group, the experimental group, hair growth was clearly observed after the 14th day of the experiment, and, in particular, the individual hair growth differences were fewer and more uniform. On day 21, in the case of the negative control group, the hair growth area was smaller than the other experimental groups. On the other hand, the FINA group, which is a positive control group, showed the fastest hair growth effect compared to other experimental groups. The dorsal skin of each mouse was photographed with constant condition by fixing the magnification and brightness of dermoscope. The relative hair density of the HR group was significantly 2.84 times lower than NOR group. By treating with 50 mg/kg and 150 mg/kg YH complex, hair regrowth rate was increased by about 117.9% and 181.5% more than the HR group, respectively. Similarly, relative hair density of the FINA group was increased by about 159.9% more than the HR group, which was higher than the YH 50 group but was lower than the YH 150 group (Figure 3A).

### 3.4. Effects of YH Complex on Hair Shaft Morphology

The surface and diameter of the hair sample were observed under the scanning electron microscope. The cracks on the cuticle of the hair surface obviously appeared in the depilated mice of the HR group. There were recoveries on the cuticle of the hair shaft body in the YH-complex-treated groups. In addition, the average diameter in the HR group was relatively (31.8%) narrow compared to that in the NOR group. The YH complex treatment dose-dependently increased the hair diameter after depilation. Especially, 150 mg/kg of YH complex administration significantly increased the cross-section diameter of the hair shaft by about 40.2% compared to the nontreated HR group (Figure 3B).

### 3.5. Effects of YH Complex on Hair Follicles in the Anagen Phase

**Figure 4:**
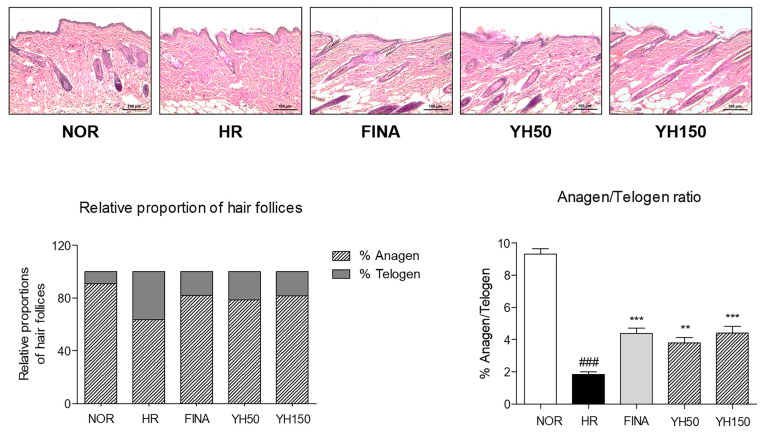
Histological changes of hair follicles of dorsal skin tissues. Hair regrowth of dorsal skin tissues monitored by H&E staining. The number of anagen and telogen phase hair follicles was counted, and then the relative proportion of hair follicles and anagen/telogen ratio were quantified. Results are presented as mean ± standard error of the mean. ^###^ *p* < 0.001 vs. NOR group; ** *p* < 0.01 and *** *p* < 0.001 vs. HR group. HR, hair removal; FINA, finasteride; YH, YH complex.

We performed H&E staining on tissue samples from these mice to analyze the telogen and anagen phases of the negative control group, positive control group, and the YH complex group. In the case of the negative control group, hair follicles decreased by 42.6% compared to the normal group, reducing the rate of anagen. The control samples were still in the telogen phase, but the FINA and YH-complex-treated samples entered the anagen phase and increased the number of hair matrix cells (Figure 4). As a result of quantitative analysis, it was confirmed that the anagen/telogen ratio increased over time after hair removal, and significant hair follicles were observed in the FINA and combination treatment group compared to the control group.

### 3.6. Effects of YH Complex on Keratinocyte by IHC Analysis

**Figure 5:**
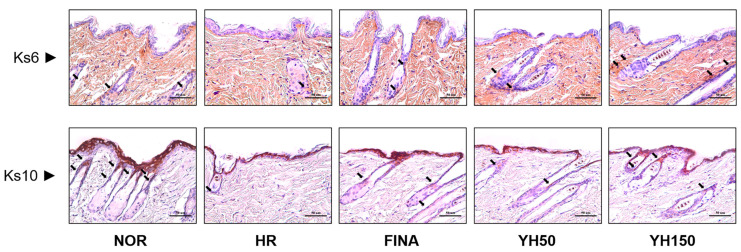
The expression of keratin in the hair follicle by IHC staining. Black arrows indicate each expression of Ks6 and Ks10. HR, hair removal; FINA, finasteride; YH, YH complex.

The YH complex increased the expression of Ks6 and Ks10 in the epidermis to a degree comparable to finasteride treatment (Figure 5). The YH complex also increased inner and outer hair follicle expression of keratinocytes, which is a key molecule in tissue repair and anagen induction in the dorsal skin lesions of C57BL/6 mice. As expected from the histological abnormalities seen in the epidermis of hair removal mice, keratinocyte differentiation was impaired. Staining of skin with antibodies against the basal keratins Ks6 and Ks10 revealed strongly reduced levels of these keratins in basal keratinocytes of negative control mice compared with normal animals.

### 3.7. Effects of YH Complex on Hair Growth Factors in Skin

**Figure 6:**
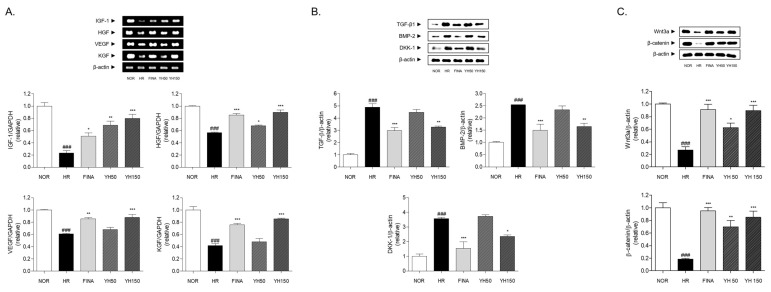
The expressions of growth factors and signaling-pathway-related molecules in skin tissues. (**A**) The mRNA expressions of growth factors including IGF-1, HGF, VEGF, and KGF. (**B**) The protein expressions of BMP-2/TGF-β signaling pathway and DKK-1. (**C**) The protein expressions of Wnt/β-catenin signaling pathway. Results are presented as mean ± standard error of the mean. ^###^ *p* < 0.001 vs. NOR group; * *p* < 0.05, ** *p* < 0.01, and *** *p* < 0.001 vs. HR group. HR, hair removal; FINA, finasteride; YH, YH complex.

It is known that hair growth promotors such as insulin-like growth factor (IGF)-1, hepatocyte growth factor (HGF), vascular endothelial growth factor (VEGF), and keratinocyte growth factor (KGF) play an important role in regulating hair reproduction. The expression level of those hair growth factors was analyzed by conducting RT-PCR (Figure 6A). The treatment of YH complex induced the mRNA level of IGF-1, HGF, VEGF, and KGF in skin tissue, compared to the control group. Specifically, in the case of HGF, VEGF, and KGF, the mRNA level was increased as YH complex concentration increased. Moreover, the mRNA levels of hair growth promotor in 150 mg/kg YH-treated group were significantly increased compared to the mRNA level in the finasteride-treated group.

### 3.8. Effects of YH Complex on Hair Growth Inhibitor in Skin

The hair growth inhibitor, including TGF-β1, BMP-2, and DKK-1, controls keratinocyte growth on dermal papillae, suppressing hair growth rate. These factors inhibit hair follicle differentiation by regulating Wnt target genes with TGF-β1 and BMP-2 and blocking β-catenin expression by DKK-1. The expression level of the hair growth inhibitor was measured by Western blotting. By hair removal, the expression levels of the hair growth inhibitor were increased. However, hair growth inhibitor expressions were dramatically downregulated by treating with a high concentration of YH complex and finasteride (Figure 6B).

### 3.9. Effects of YH Complex on Wnt/β-Catenin Signaling Pathway in Skin

Hair removal led to a reduction in Wnt3a expression in the skin tissues, compared to skin tissues of the NOR group. The expression of β-catenin is regulated by Wnt3a. The YH complex treatment (50 and 150 mg/kg) increased the Wnt3a by about 135.2 and 236.7%, respectively. The FINA group showed a 242.4% increase in Wnt3a protein expression. Additionally, the protein expression of β-catenin was significantly increased in YH 50, YH150, and FINA groups (Figure 6C).

## 4. Discussion

Hair follicles have multidifferentiation capacity and proliferation potential due to the circulatory process [29]. These abilities primarily contribute to the high regenerative capacity of hair and repair of skin [8]. The growth of the hair follicle is divided into three phases: anagen, a growing stage; catagen, an apoptosis-mediated regression stage; and telogen, a resting stage [30]. Especially, recent studies found that telogen is a main controlling phase of the hair follicle cycle [29]. It is not just an inactive state; rather, it is a period to build up energy for a rapid response to hair loss [31]. After that, the size of the hair follicle is extended and a hair shaft with hair fiber is formed during the anagen phase [32]. In that stage, several signaling pathways are involved in the differentiation of the hair follicle to make them promote regenerating and reprogramming of hair formation [33]. Hence, the activation of these signaling-pathways-related mediators and factors may lead to hair growth against hair loss or hair formation obstacles [34]. In this study, the YH complex, a newly developed formulation consisting of *A*. *membranaceus* and *C*. *cassia*, was investigated on hair growth and its related signaling pathways in depilated hair removal mice. Prior to testing hair growth effects of the YH complex, the potential molecular mechanism of the YH complex was predicted through network pharmacological analysis. Network pharmacology is regarded as an emerging tool to explore potential drug-constructing complex relationships between herbs, compounds, target genes, and diseases [35]. We found that the gene set of *A*. *membranaceus* and *C*. *cassia* was about 74.8% overlapped with the gene set of the ‘Hair growth’ database, indicating that there is a high association with the potential of the YH complex on hair growth. From functional enrichment analysis on the GO Biological Process database, ‘Cellular response to growth factor stimulus’, ‘Response to growth factor’, ‘Regulation of hair cycle’, ‘Hair follicle development’, and ‘Hair cycle’ were predicted as significant target pathways of the YH complex. From those results, we assumed the hair growth effects of YH complex with the stimulation of growth factor.

Anagen-like hair follicles appear slender and straight in the penetrated state into the subcutaneous tissue [36]. During that stage, the morphology of hair shaft is complete and hair fiber grows rapidly, while telogen-like hair follicles are weak and short without the formation of hair fiber [30]. In the present study, oral administration of the YH complex promoted hair regrowth in depilated hair loss mice. Hair density on the dorsal skin monitored by dermoscope was increased by the YH complex compared to nontreated hair loss mice. In addition, SEM analysis showed a significant increased hair shaft thickness at a normal level. The cracks on the cuticle of the hair surface were obviously recovered by YH complex treatment for 3 weeks. From the histological result, the relative proportion of hair follicles in % anagen and % telogen was varied by the YH complex. The anagen/telogen ratio of 150 mg/kg of YH complex-treated mice was significantly increased. Taken together, we found that the YH complex promoted hair growth and regulated the hair cycle into the anagen phase in hair loss mice.

**Figure 7:**
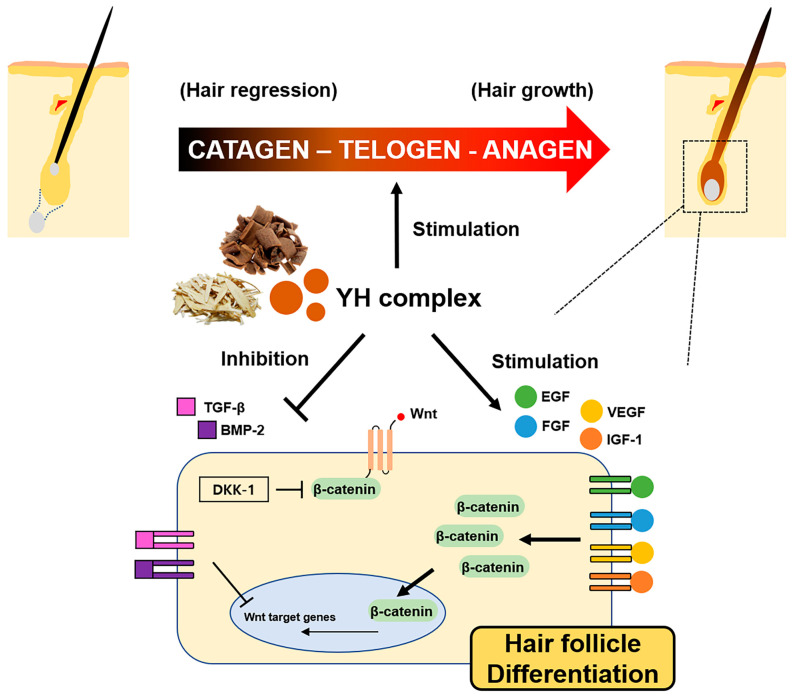
The diagram of molecular action of the YH complex on hair growth.

Numerous signaling pathways interact with one another by forming a regulatory complex crucial for the growth and maintenance of hair [37]. In particular, the Wnt/β-catenin signaling pathway is considered the main target for hair follicles development, transitioning from the resting telogen stage to the growing anagen stage [34]. It plays a vital role in the process of hair follicles differentiation with the regulation of various cytokines and growth factors [38]. The growth factors, including IGF-1, HGF, VEGF, and KGF, affect the growth of hair follicles, and are involved in the regulation of hair morphogenesis and the hair cycle. IGF-1 secretion in a paracrine manner in the dermis induces epidermal cell proliferation and participates in the development of skin [39]. Additionally, IGF-1 has been found to inhibit apoptotic factors in the regression–catagen phase [40]. It is known that HGF and VEGF have great importance in the development and differentiation of hair. There are reports that HGF stimulates the development of hair follicle in mouse vibrissae and human scalp hairs [41]. It induces and preserves the anagen phase in hair follicles of mice. HGF and VEGF promote the hair growth by perifollicular vascularization and angiogenesis [42]. On the other hand, KGF, also as known as FGF-7, has been known to stimulate the elongation of hair fibers and inhibit the transition from anagen to catagen of the hair cycle [43]. The mRNA expressions of those growth factors including IGF-1, HGF, VEGF, and KGF were dose-dependently increased by the treatment of the YH complex. The elevated growth factors interact with the release of β-catenin in hair growth or hair loss; likewise, DKK-1 inhibits Wnt action in hair follicles [44]. Separated β-catenin from the Wnt/β-catenin complex translocates to the nucleus to initiate the regeneration of hair follicles and induce the onset of anagen phase [45]. During these processes, the AKT/PI3K signaling pathway and the MAP kinase signaling pathway are simultaneously involved in the phosphorylation of GSK3β, resulting in the promotion of β-catenin release [44]. The Wnt and β-catenin expressions in skin tissues were apparently increased, while the DKK-1 expression was declined by the administration of the YH complex. Moreover, studies suggested that the BMP/TGF-β signaling pathway inhibited the hair growth [46]. BMP-2 is expressed in the late growth period, thus acerbating the transition to catagen phase [47]. The YH complex effectively inhibited the expressions of BMP-2 and TGF-β in the skin of depilated hair loss mice. These results demonstrate that the growth factors elevated by the YH complex induced β-catenin translocation into the nucleus, leading to the hair follicle differentiation and development in hair loss mice. The inhibitory properties of the BMP/TGF-β signaling pathway against hair growth were decreased by the YH complex. Eventually, the YH complex exhibited hair-growing effects via promotion of the Wnt/β-catenin signaling pathway and inhibition of the BMP/TGF-β signaling pathway, thereby increasing the expression of keratin 6 and 10, indicators of hair growth, and the formation of anagen hair follicles (Figure 7).

## 5. Conclusions

In conclusion, the YH complex, consisting of *A*. *membranaceus* and *C*. *cassia*, induced the hair follicles’ differentiation and preserved the growing anagen phase, revealing the hair-growth-promoting property. Our study demonstrated that the increases of growth factors, including IGF-1, HGF, VEGF, and KGF, and decreases of DKK-1 and BMP/TGF-β signaling pathways by the YH complex promoted the activation of the Wnt/β-catenin signaling pathway to transit and maintain the growing anagen stage in hair loss. The YH complex could be a novel remedy for hair loss diseases such as alopecia areata, androgenetic alopecia, telogen effluvium, and chemotherapy-induced alopecia.

## Data Availability

All data are available from the corresponding authors upon reasonable request.

## Supplementary Materials

The following supporting information can be downloaded at: https://www.mdpi.com/article/10.3390/cimb45110541/s1, Figure S1: Identification of YH complex using standards of cinnamic acid and calycosin-7-O-glucoside. Table S1. Target genes of *Cinnamomum cassia* on TM-MC database. Table S2. Target genes of *Astragalus membranaceus* on TM-MC database. Table S3. Chemical compounds of *Cinnamomum cassia* and their related genes. Table S4. Chemical compounds of *Astragalus membranaceus* and their related genes. Table S5. Total genes of *Cinnamomum cassia*. Table S6. Total genes of *Astragalus membranaceus*. Table S7. YH complex network; sum of related genes of *Cinnamomum cassia* and *Astragalus membranaceus*. Table S8. Total genes related to hair growth from HUMANBASE database.
