# Supplementary figures and images for: Astragalus membranaceus and Cinnamomum cassia Stimulate the Hair Follicle Differentiation-Related Growth Factor by the Wnt/β-Catenin Signaling Pathway
Source: Curr Issues Mol Biol. 2023 Oct 26;45(11):8607–21. doi: 10.3390/cimb45110541 (PMC10670826; doi:10.3390/cimb45110541)

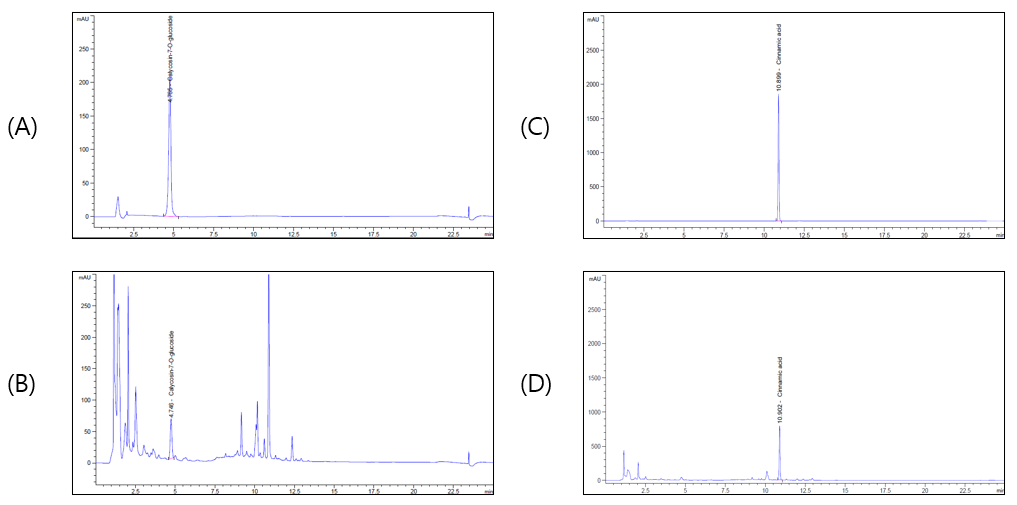

Supplement: Supplementary file 1 [file cimb-45-00541-s001.zip › Supplementary figure.tif]
